# Spatial regulation of benzylisoquinoline alkaloid biosynthesis in lotus (*Nelumbo nucifera*) is controlled coordinately through the NnMYC2-NnMYB14-NnCYP80 modules

**DOI:** 10.1093/hr/uhaf283

**Published:** 2025-10-16

**Authors:** Minghua Zhang, Zhiting Xu, Zijian Yang, Heyun Song, Jia Xin, Hui Yang, Heng Sun, Juan Liu, Dong Yang, Yanling Liu, Jing Li, Mei Yang, Xianbao Deng

**Affiliations:** State Key Laboratory of Plant Diversity and Specialty Crops, Wuhan Botanical Garden, Chinese Academy of Sciences, Wuhan 430074, China; University of Chinese Academy of Sciences, Beijing 100049, China; School of Chemistry, Chemical Engineering and Life Sciences, Wuhan University of Technology, Wuhan 430070, China; School of Chemistry, Chemical Engineering and Life Sciences, Wuhan University of Technology, Wuhan 430070, China; State Key Laboratory of Plant Diversity and Specialty Crops, Wuhan Botanical Garden, Chinese Academy of Sciences, Wuhan 430074, China; University of Chinese Academy of Sciences, Beijing 100049, China; State Key Laboratory of Plant Diversity and Specialty Crops, Wuhan Botanical Garden, Chinese Academy of Sciences, Wuhan 430074, China; University of Chinese Academy of Sciences, Beijing 100049, China; State Key Laboratory of Plant Diversity and Specialty Crops, Wuhan Botanical Garden, Chinese Academy of Sciences, Wuhan 430074, China; University of Chinese Academy of Sciences, Beijing 100049, China; State Key Laboratory of Plant Diversity and Specialty Crops, Wuhan Botanical Garden, Chinese Academy of Sciences, Wuhan 430074, China; Hubei Key Laboratory of Wetland Evolution & Ecological Restoration, Wuhan Botanical Garden, Chinese Academy of Sciences, Wuhan 430074, China; State Key Laboratory of Plant Diversity and Specialty Crops, Wuhan Botanical Garden, Chinese Academy of Sciences, Wuhan 430074, China; Hubei Key Laboratory of Wetland Evolution & Ecological Restoration, Wuhan Botanical Garden, Chinese Academy of Sciences, Wuhan 430074, China; State Key Laboratory of Plant Diversity and Specialty Crops, Wuhan Botanical Garden, Chinese Academy of Sciences, Wuhan 430074, China; Hubei Key Laboratory of Wetland Evolution & Ecological Restoration, Wuhan Botanical Garden, Chinese Academy of Sciences, Wuhan 430074, China; State Key Laboratory of Plant Diversity and Specialty Crops, Wuhan Botanical Garden, Chinese Academy of Sciences, Wuhan 430074, China; Hubei Key Laboratory of Wetland Evolution & Ecological Restoration, Wuhan Botanical Garden, Chinese Academy of Sciences, Wuhan 430074, China; School of Chemistry, Chemical Engineering and Life Sciences, Wuhan University of Technology, Wuhan 430070, China; State Key Laboratory of Plant Diversity and Specialty Crops, Wuhan Botanical Garden, Chinese Academy of Sciences, Wuhan 430074, China; Hubei Key Laboratory of Wetland Evolution & Ecological Restoration, Wuhan Botanical Garden, Chinese Academy of Sciences, Wuhan 430074, China; State Key Laboratory of Plant Diversity and Specialty Crops, Wuhan Botanical Garden, Chinese Academy of Sciences, Wuhan 430074, China; Hubei Key Laboratory of Wetland Evolution & Ecological Restoration, Wuhan Botanical Garden, Chinese Academy of Sciences, Wuhan 430074, China

## Abstract

Plant benzylisoquinoline alkaloids (BIAs) are a group of plant-specialized metabolites with significant pharmacological properties. In lotus (*Nelumbo nucifera*), BIAs accumulate primarily in the leaf blade and plumule organs. The two organs, however, accumulate quite different types of BIAs, within the former primarily aporphine-type BIAs, while the latter predominantly bis-BIAs. Herein, we demonstrate that the spatial regulation of BIA biosynthesis in lotus is coordinately controlled through the NnMYC2-NnMYB14-NnCYP80 modules. Genome-wide screening of lotus *CYP80* genes discovered two tandemly arrayed yet tissue-specific *NnCYP80s* that are identical to the previously reported *NnCYP80G* and *NnCYP80A*, respectively. *NnCYP80G* is expressed primarily in the lotus laminae, while *NnCYP80A* is expressed particularly in the plumules. Our enzyme assays confirmed the proaporphine synthase activity of NnCYP80G and the bis-BIA synthase activity of NnCYP80A, and revealed the aporphine synthase activity of NnCYP80G by efficiently converting the (*R*)-reticuline substrate into corytuberine. In addition, we characterized an R2R3 MYB transcription factor (TF) NnMYB14, which binds directly to the *NnCYP80G* and *NnCYP80A* promoters and positively regulates their expression. NnMYC2, the core regulator in the JA signaling pathway, acts very upstream of NnMYB14, by binding directly to the *NnMYB14* promoter and inducing its expression. Our results resolved that the organ-specific accumulation of BIAs in lotus is attributed to the tissue-specially expressed *NnCYP80G* and *NnCYP80A* genes, and the NnMYC2-NnMYB14 TF module could positively regulate the *NnCYP80G* and *NnCYP80A* expression and the lotus BIA biosynthesis.

## Introduction

Under stressful circumstances, plants produce a large array of secondary metabolites, which contribute to plant color, flavor, fragrance, and impart protective barriers through a variety of antimicrobial, pesticidal, phytoalexin, and UV filter properties [1, 2]. Due to the unique biological functions and inherent cytotoxicities, secondary metabolites accumulate frequently in tissue- or organ-specific patterns. Typically, anthocyanin pigments accumulate primarily in floral organs to attract pollinators, whereas aromatic terpenoids are often present in fruits to lure animals for seed dispersal [3–5]. The tissue-specific localization of secondary metabolites is believed to be attributed to the spatial accumulation of the biosynthetic enzymes or transporters [6]. However, localization and functional characterization of such enzymes in most secondary metabolite pathways remain to be determined comprehensively.

Benzylisoquinoline alkaloids (BIAs) are a class of tyrosine-derived plant metabolites, with >2500 characterized chemical structures [7, 8]. These molecules play important roles in plant defense against herbivores and pathogens, while also possessing significant pharmacological properties [9–12]. Typically, the BIA morphine and codeine are potent analgesics, noscapine possesses potential anticancer activities, and sanguinarine and berberine are excellent antimicrobial agents [13]. In contrast to the phenylpropanoids and terpenoids, which generally occur in higher plants, BIAs are restricted to certain families, primarily in the orders of Ranunculales and Magnoliales, but are also found in the distantly related families of Rutaceae, Lauraceae, Cornaceae, and Nelumbonaceae [14]. Research on the BIA biosynthesis has been focused on a limited number of plant species, mainly in the opium poppy (*Papaver somniferum*), California poppy (*Eschscholzia californica*), and Japanese goldthread (*Coptis japonica*). These species are emerging models for investigating the biosynthesis and regulation of plant BIAs.

The biosynthesis of BIAs involves at least five P450-mediated reactions, including hydroxylation (CYP80B) [15], methylenedioxy bridge formation (CYP719A) [16, 17], and phenol-coupling reactions (CYP80A, CYP80G, and CYP719B) [18]. Of these reactions, CYP80A catalyzes the intermolecular C-O phenol coupling during the formation of bis-benzylisoquinoline alkaloids (bis-BIAs) [19], whereas CYP80G mediates an intramolecular C-C phenol-coupling reaction in the aporphine-type BIA biosynthesis. Despite performing distinct catalytic functions, CYP80A, CYP80B, and CYP80G share rather high DNA and amino acid sequence similarities, and are considered to be derived from a common ancestor [20–22].

**Figure 1:**
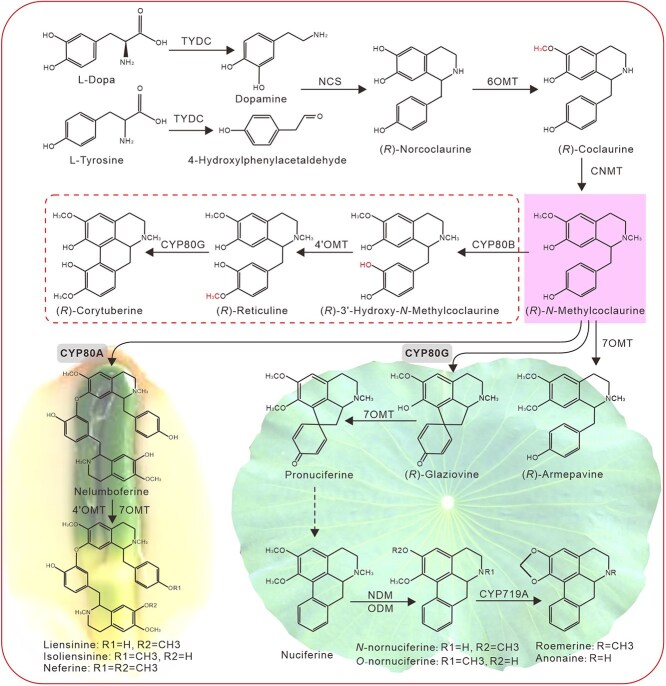
NnCYP80A and NnCYP80G (marked with grey background) are the key enzymes involved respectively in the lotus plumule and lamina BIA biosynthesis. Branched steps framed with broken lines are probably missed in lotus. (*R*)-*N*-Methylcoclaurine (marked with purple background) serves as the central intermediate and branch point in the biosynthesis of bis-BIAs and aporphine BIAs.

Lotus (*Nelumbo nucifera*) is the most widely cultivated aquatic crop in China, possessing significant commercial values, for its magnificent flowers, tasty seeds, and nutritious rhizomes [23]. Lotus organs are also rich in pharmacologically significant BIAs. To date, >50 BIA structures have been characterized in lotus, primarily in its lamina and plumule organs [24–27]. In contrast to the predominantly found *S*-enantiomers in other species, BIAs detected in lotus are mostly *R*-enantiomer conformations [25]. According to chemical structures, lotus BIAs can be classed into three subgroups, the 1-benzylisoquinolines, the aporphines, and the bis-BIAs. Aporphines and bis-BIAs are the main products detected in lotus, accounting for >99% of the total alkaloids in the laminae and plumules [24]. By contrast, 1-benzylisoquinolines are generally intermediates during aporphine and bis-BIA production, and generally accumulate in trace amounts. Intriguingly, aporphine-type BIAs, including nuciferine, *N*-nornuciferine, *O*-nornuciferine, roemerine, and anomaine, occur predominantly in the lotus laminae and petals, whereas bis-BIAs, such as liensinine, isoliensilnine, and neferine, were found primarily in the plumules (Fig. 1) [24, 28, 29]. According to the deduced lotus BIA biosynthesis pathway, NnCYP80G and NnCYP80A are the key enzymes responsible for the biosynthesis of aporphine-type BIAs and bis-BIAs, respectively (Fig. 1) [30]. The two enzymes, also named as NnCYP80Q1 and NnCYP80Q2 [31, 32], were recently identified, based on their amino acid sequence similarities to previous reported CYP80 enzymes [21, 33, 34]. The yeast-expressed NnCYP80G and NnCYP80A were able to catalyze the biosynthesis of a proaporphine and a bis-BIA, respectively. In addition, lotus BIA biosynthesis was inducible by mechanical wounding and methyl jasmonate (MeJA) treatment, and JA-responsive transcription factors (TFs), including WRKYs, ERFs, bHLHs, and MYBs, are potential regulators of lotus BIA biosynthesis [24, 30, 35, 36]. However, the *in planta* functions, (*R*)-substrate preference, and underlying regulatory networks of NnCYP80G and NnCYP80A remain to be elucidated.

In this study, we performed genome-wide screening of lotus cytochrome P450 genes and identified two tissue-specific lotus *CYP80* genes, *NnCYP80G* and *NnCYP80A*, with the former expressed primarily in the lotus laminae, while the latter particularly in the plumules. Enzyme activity assays and lotus petal overexpression data revealed that NnCYP80G is involved in the biosynthesis of aporphine-type BIAs, whereas NnCYP80A catalyzed the formation of bis-BIAs. We also characterized a JA-responsive NnMYC2-NnMYB14 module, which positively regulates the lotus BIA biosynthesis by directly binding to and activating both *NnCYP80* genes. Our evidence revealed for the first time that the spatial regulation of BIA biosynthesis in lotus is controlled coordinately by the NnMYC2-NnMYB14-NnCYP80G/NnCYP80A modules.

## Results

### Mining of CYP80s involved in the lotus BIA biosynthesis

**Figure 2:**
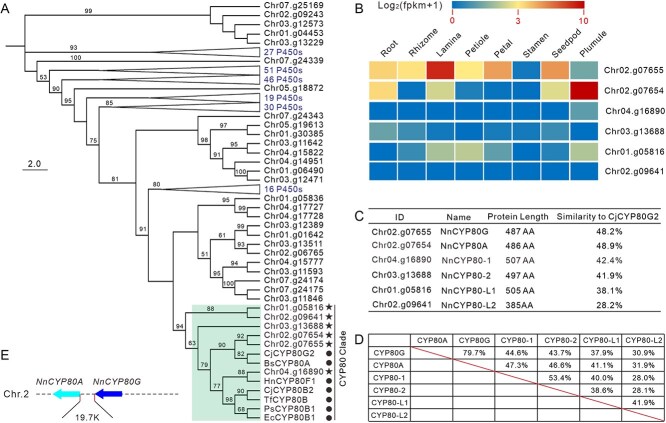
Phylogeny, sequence characteristic, and transcript profiling of the lotus *CYP80* gene family. (A) An NJ phylogenetic tree based on the full-length amino acid sequences of 222 lotus P450 proteins and seven previously identified plant CYP80s that involved in BIA biosynthesis. The tree was constructed in MEGA7 with 1000 bootstrap replications. The CYP80 clade and lotus CYP80 are marked with pentagrams, while the CYP80 references from other species are marked with filled circles. (B) Spatial expression patterns of *NnCYP80* genes. (C) The lotus CYP80 naming, protein length, and amino acid sequence similarity vs CjCYP80G2. (D) Amino acid sequence similarities between NnCYP80s. (E) Schematic presentation of the tandemly arrayed *NnCYP80G* and *NnCYP80A* on chromosome 2 of the lotus genome.

To identify potential CYP80s involved in the lotus BIA biosynthesis, we conducted a genome-wide investigation of lotus P450 genes. By screening our unpublished lotus telomere-to-telomere (T2T) genome assembly (Sun *et al*., unpublished data), we identified a total of 222 lotus P450 genes (Supplementary Data S1). Of these *P450s*, six were phylogenetically clustered with the seven characterized BIA CYP80 reference genes, thus were deemed to be potential lotus CYP80s (Fig. 2A). Among these six NnCYP80s, Chr02.g07654 and Chr02.g07655 were clustered with the *C. japonica* CYP80G2 and *Berberis stolonifera* CYP80A, Chr04.g16890 fell within the plant CYP80B clade, while the remaining three were adjacent to the core plant CYP80s. To verify whether these three lotus CYP80s are true CYP80s, a second phylogenetic tree was further constructed. This tree included CYP80s of 20 BIA producing Ranunculales species [37], as well as putative CYP80 or CYP80-like proteins of 10 plant species, selected to represent different positions of plant phylogeny. These 10 species are: *Physcomitrella patens* (mosses), *Selaginella moellendorffii* (ferns), *Pinus taeda* (gymnosperms), *Amborella trichopoda* (the most basal flowering plant), *Oryza sativa* and *Musa acuminate* (monocots), *Nymphaea colorata*, *N. nucifera*, *Arabidopsis thaliana*, and *Solanum lycopersicum* (dicots) (Fig. S1A and B; Supplementary Data S2). Intriguingly, all CYP80s of the 20 BIA-producing Ranunculales species were clustered together, forming a core CYP80 clade. Four of the six putative lotus CYP80s were located in this clade, thus were deemed to be true lotus CYP80s. Interestingly, CYP80s within this clade also included proteins of water lily and tomato, which probably produce BIAs [14, 38, 39].

Based on our previously conducted transcriptomic data, only two of the six *NnCYP80*s (*Chr02.g07654* and *Chr02.g07655*) are expressed in the lotus cv. ‘China Antique’. Markedly, the *Chr02.g07655* was expressed primarily in the lotus laminae, while the *Chr02.g07654* was expressed predominantly in the lotus plumules (Fig. 2B). This matched well to the accumulation patterns of aporphines and bis-BIAs, which deposited mainly in the lotus laminae and plumules, respectively [24]. According to these evidences, we designated the *Chr02.g07655* and *Chr02.g07654* as *NnCYP80G* and *NnCYP80A*, respectively, while the rest two core CYP80 members, *Chr04.g16890* and *Chr03.g13688*, as *NnCYP80-1* and *NnCYP80-2* (Fig. 2C). The *Chr01.g05816* and *Chr02.g09641* that clustered outside the core CYP80 clade were named as *NnCYP80-like1* and *NnCYP80-like2*. It is worth noting that *NnCYP80G* and *NnCYP80A* are identical to the recently reported *NnCYP80Q1* and *NnCYP80Q2* [31], respectively.

The four NnCYP80s shared 41.9%–48.9% amino acid similarity with CjCYP80G2 [21], with their protein lengths varying from 486 to 507 amino acids (Fig. 2C). The similarities between lotus CYP80s were relatively higher, up to 79.7% between the NnCYP80G and NnCYP80A (Fig. 2D). Interestingly, *NnCYP80G* and *NnCYP80A* are two genes tandemly arrayed on the minus strand of lotus chromosome 2, with an ~19-kb interval and zero intervening spacer genes between them (Fig. 2E). Synteny analysis of lotus P450s revealed a total of 31 paralog pairs (Fig. S2), indicating the contribution of large-scale gene duplications or whole-genome duplications (WGDs) on the lotus P450 super family expansion. The six putative *NnCYP80* genes, however, displayed no collinearity relationships with each other. Thus the lotus *CYP80* gene family was expanded mainly through small-scale gene duplication events, such as unequal chromosomal crossing-over or retroposition [40].

### The *NnCYP80G* and *NnCYP80A* expression was positively correlated with BIA accumulations in the lotus laminae and plumules, respectively

**Figure 3:**
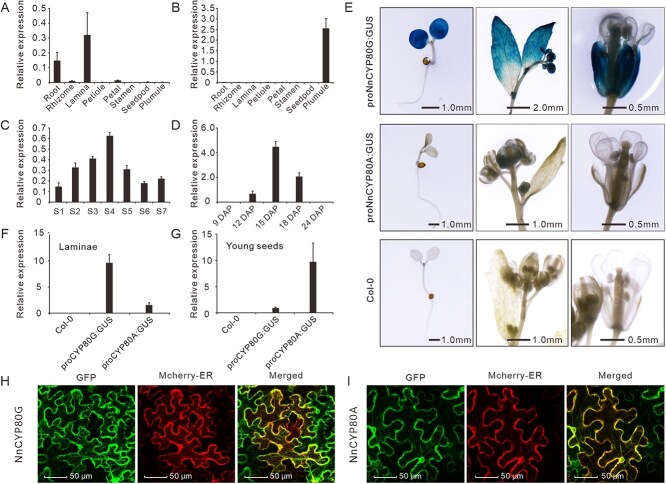
The expression patterns of *NnCYP80G* and *NnCYP80A*. (A) and (B) *NnCYP80G* and *NnCYP80A* expression in the eight lotus organs. (C) *NnCYP80G* expression in the seven lamina developmental stages. (D) *NnCYP80A* expression in the five plumule developmental stages. (E) GUS staining in the *proNnCYP80G:GUS* and *proNnCYP80A:GUS* transgenic *Arabidopsis*. (F and G) Quantitative real-time PCR checking the *GUS* expression in the leaves and young seeds of *proNnCYP80G:GUS* and *proNnCYP80A:GUS* transgenic *Arabidopsis*. (H) and (I) Subcellular localization of NnCYP80G and NnCYP80A in the infiltrated *N. benthamiana* cells. Panels from left to right refer to the NnCYP80G:GFP (or NnCYP80A:GFP), the ER marker protein Mcherry-ER, and the merged images, respectively. Data are means ± SE (*n* = 3).

Next, we evaluated further the expression patterns of *NnCYP80G* and *NnCYP80A* through real-time quantitative polymerase chain reaction (RT-qPCR). Spatially, *NnCYP80G* was mainly expressed in the lotus laminae and roots, followed by the petal, rhizome, and seedpod organs (Fig. 3A). In contrast, *NnCYP80A* was expressed particularly in the lotus plumules (Fig. 3B). As *NnCYP80G* and *NnCYP80A* were expected to be involved in the biosynthesis of the lamina aporphine BIAs and the plumule bis-BIAs, we also checked their temporal expression in different lamina and plumule developmental stages. As a result, *NnCYP80G* expression in the lotus lamina was low at the bud stage (Developmental Stage 1, S1), gradually increased during the early developmental stages, peaked at Stage 4 (S4), and then steadily decreased during the late stages (Fig. 3C). The expression of *NnCYP80A* in lotus plumules was almost undetectable before 9 and after 24 days after pollination (DAP), but was high between 12 and 18 DAP, with a peak value at 15 DAP (Fig. 3D). The *NnCYP80G* and *NnCYP80A* expression profiles correlated well to our previously reported aporphine BIA and bis-BIA accumulation patterns [24, 41], indicating further their potential functions in the lotus BIAs biosynthesis.

The expression patterns of *NnCYP80G* and *NnCYP80A* were also verified in the *proNnCYP80G:GUS* and *proNnCYP80A:GUS* transgenic *Arabidopsis*. Promoter regions ~1.5 kb of the two genes were isolated and fused to the *β-glucuronidase* (GUS) reporter gene for the construction of *proNnCYP80G:GUS* and *proNnCYP80A:GUS* plasmids, and the following *Arabidopsis* (Col-0) transformation. The histochemical assays detected intensive GUS in all the green tissues of *proNnCYP80G:GUS* transgenic *Arabidopsis*, including cotyledons, true leaves, and flower bracts (Fig. 3E). In contrast, the *proNnCYP80A* driven *GUS* was detected only in the young seeds of *proNnCYP80A:GUS* transgenic *Arabidopsis* (Fig. 3F and G), which was consistent with the spatial expression patterns of *NnCYP80G* and *NnCYP80A* in the lotus tissues.

Protein structure analysis indicated that all the four lotus CYP80 proteins harbored conserved eukaryotic P450 regions, including the helix K, aromatic, and heme-binding regions at the C-terminal end (Fig. S3). In addition, the N-terminal of these proteins contained hydrophobic domains corresponding to the membrane anchor sequences of microsomal P450 species, suggesting their endoplasmic reticulum (ER) localization. The subcellular localization assay conducted on *Nicotiana benthamiana* leaves confirmed their ER localizations. Both NnCYP80G:GFP and NnCYP80A:GFP were colocalized with the ER marker (Mcherry) (Fig. 3H and I). Similar as CjCYP80G2, both NnCYP80G and NnCYP80A had an amino acid substitution in the consensus helix I region [(A/G)G*X*(D/E)T(T/S)], with a proline instead of alanine/glycine residue at the position corresponding Gly-248 in P450cam [42]. In contrast, this single amino acid substitution, which is involved in the interaction with the substrate and iron-bound oxygen, was absent in the other four lotus CYP80s.

### The functions of NnCYP80G and NnCYP80A were diverged in the lotus BIA biosynthesis

**Figure 4:**
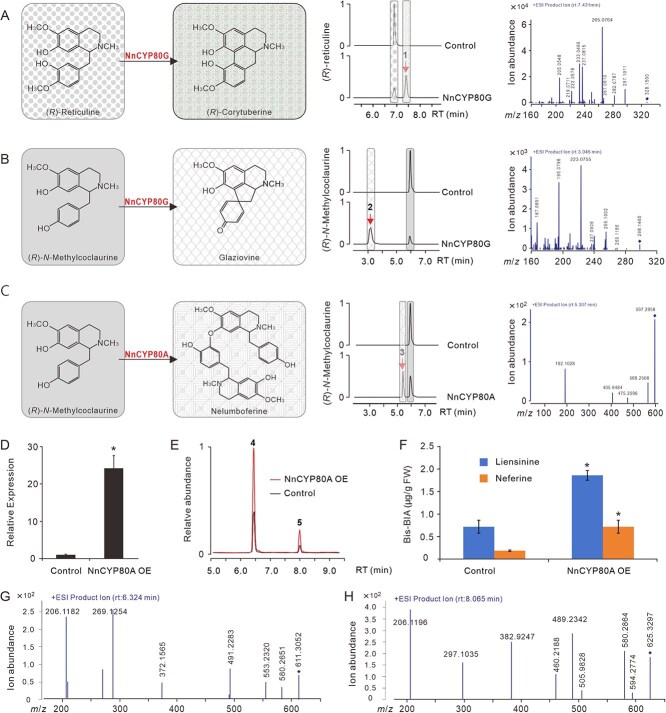
Identification of phenol-coupling activities of NnCYP80G and NnCYP80A in the *N. benthamiana* leaves. (A–C) Catalytic reactions of NnCYP80G and NnCYP80A fed with different 1-benzylisoquinoline substrates, the corresponding extracted ion chromatograms (EIC), and the MS/MS spectrum of product peaks. (A) NnCYP80G + (*R*)-reticuline (marked with grey sugar background); (B) NnCYP80G + (*R*)-*N*-methylcoclaurine (marked with grey background); and (C) NnCYP80A + (*R*)-*N*-methylcoclaurine. Products of (*R*)-corytuberine, glaziovine, and nelumboferine were marked with concrete, net, and curtain backgrounds, respectively. (D–F) *NnCYP80A* expression (D) and the accumulation of bis-BIAs (E and F) on the lotus petals overexpression of *NnCYP80A*. (G) and (H) The MS/MS spectrum of liensinine and neferine. Peaks 1–5 represent (*R*)-corytuberine, glaziovine, nelumboferine, liensinine, and neferine, respectively. Data are means ± SE (*n* = 3), and asterisks indicate statistically significant difference determined by two-tailed *t*-test (*P* ≤ 0.05).

Functions of NnCYP80G and NnCYP80A have been previously characterized in the yeast BIA platforms [31, 34]. In the engineered yeast, NnCYP80G was able to convert (*R*)-N-methylcoclaurine to the proaporphine glaziovine, while NnCYP80A also efficiently took the (*R*)-N-methylcoclaurine substrate, leading to the production of bis-BIA nelumboferine. To elucidate the phenol-coupling activities of the two enzymes *in planta*, the two proteins were transiently expressed in *N. benthamiana* leaves, together with a lotus cytochrome P450 reductase (*NnCPR*, Nn2g12771). Four days after agroinfiltration, the infiltrated leaves were fed with different BIA substrates, and then harvested after 24 h for ultrahigh-performance liquid chromatography-tandem mass spectrometry (UPLC-MS/MS) analysis. RT-qPCR analysis demonstrated the successful expression of the three genes, with their relative expression ~2.8–9.2 (*NnCPR*), 4265.9 (*NnCYP80G*), and 124.4 (*NnCYP80A*) times of the *NbACTIN* expression, respectively (Fig. S4A). Coexpressed NnCYP80G and NnCPR did not consume coclaurine, (*S*)-*N*-methylcoclaurine, armepavine, and (*S*)-reticuline substrates (Fig. S4B), but intensively accepted the (*R*)-reticuline (*m*/*z* 330) and (*R*)-*N*-methylcoclaurine (*m*/*z* 300) (Fig. 4A; Fig. S5A). Feeding of (*R*)-reticuline resulted in the formation of a new peak (peak **1;** RT, 7.353 min), with an ~85% conversion efficiency. This product had a mass charge of *m*/*z* [M + H]^+^ 328.1500, and its MS/MS spectrum contained main fragment ions of *m*/*z* 205.1, 222.1, 237.1, 265.1, 267.1, 282.1, and 297.1, which is identical to the previously identified corytuberine compound [21, 22] (Fig. 4A). NnCYP80G plus its redox partner also converted 56% of (*R*)-*N*-methylcoclaurine to peak **2** (RT 3.165 min). This peak is correspondent with a pronuciferine compound glaziovine, which had a mass charge of *m*/*z* 298.1465 and contained main peaks at *m*/*z* 167.1, 195.1, 223.1, 237.1, 255.1, and 269.1 in the MS/MS spectrum [34] (Fig. 4B; Fig. S5B).

The *Agrobacterium-*expressed NnCYP80A consumed only the (*R*)-*N*-methylcoclaurine substrate, with a conversion rate ~16%, leading to the production of an obvious new peak (peak **3**) (Fig. 4C; Fig. S5C). This product had a parent mass of *m*/*z* 597.2958 and retention time at 5.380 min. Its MS/MS spectrum contained main fragments of *m*/*z* 192.1, 475.2, and 566.3, which was identical to a previously reported bis-BIA of nelumboferine [34].

To characterize further the function of *NnCYP80G* and *NnCYP80A*, we conducted *Agrobacterium-*mediated transient overexpression in the lotus petals. Overexpression of *NnCYP80G* in the lotus petals was not successful, probably due to the relatively high background expression. However, the overexpression of *NnCYP80A* was evident, because its background expression was marginally low in the lotus petals (Fig. 4D). As a result, the accumulation of two bis-BIAs (peak **4** and **5**) was significantly enhanced, with a 2.88- and 2.41-fold increase for liensinine and neferine, respectively (Fig. 4E–H). Taken together, our results confirmed that the NnCYP80G was a proaporphine synthase, while the NnCYP80A functions as a bis-BIA synthase in lotus. We revealed for the first time that NnCYP80G also functions as an aporphine synthase by converting (*R*)-reticuline into corytuberine.

### NnMYC2 positively regulates *NnCYP80G* and *NnCYP80A* expression

**Figure 5:**
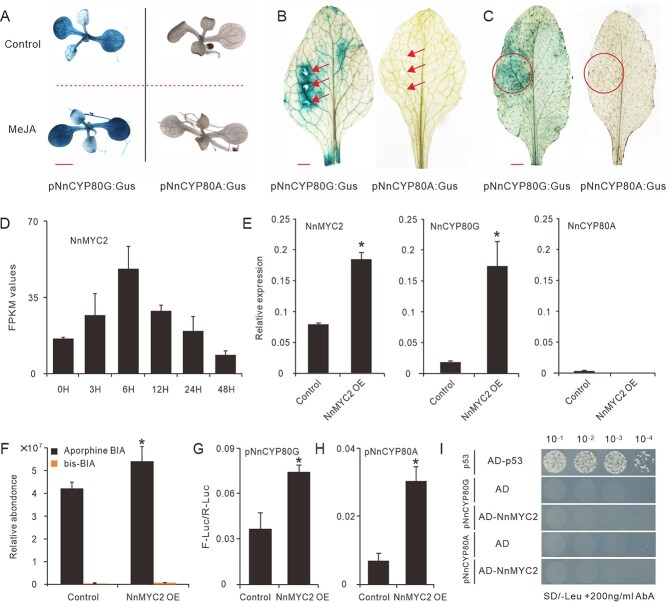
NnMYC2 regulates positively *NnCYP80G* and *NnCYP80A* expression. (A) and (B) Histochemical GUS expression assays testing the effect of MeJA (A) and wounding treatment (B) on the *proNnCYP80G:GUS* and *proNnCYP80A:GUS* expression in transgenic *Arabidopsis* leaves. (C) GUS staining testing the effect of NnMYC2 overexpression on the *NnCYP80G* and *NnCYP80A* expression. (D) *NnMYC2* expression under MeJA treatment in the lotus lamina. Digital expression was marked with FPKM values obtained from our previously conducted RNA-seq data. (E) Quantitative Real-Time PCR measured relative expression of *NnMYC2*, *NnCYP80G*, *NnCYP80A*, and *NnMYB14* in the lotus petals overexpressing *NnMYC2*. (F) The relative concentration of aporphine-BIAs and bis-BIAs in the lotus petals overexpressing *NnMYC2*. (G) and (H) Dual-luciferase assay determination the effects of NnMYC2 on the activation of *NnCYP80G* (G) and *NnCYP80A* (H) promoters. (I) Y1H assays checking the interactions between NnMYC2 and the *NnCYP80G* or *NnCYP80A* promoters. Red arrows and circles represent the wounding sites and NnMYC2 overexpression sites on the transgenic *Arabidopsis* leaves, respectively. Red bars in Panels A, B, and D indicate the length of 1 mm. Data are means ± SE (*n* = 3), and asterisks indicate statistically significant difference determined by two-tailed *t*-test (*P* ≤ 0.05).

Previously, we have shown that the BIA biosynthesis in lotus is regulated by MeJA and mechanism wounding [24, 35, 36]. To verify the likely roles of JA and wounding in regulating *NnCYP80G* and *NnCYP80A* expression, *proNnCYP80G:GUS* and *proNnCYP80A:GUS* transgenic *Arabidopsis* seedlings were subjected to MeJA and wounding treatments. As a result, both treatments resulted in much darker GUS staining in the *proNnCYP80G:GUS Arabidopsis* leaves (Fig. 5A and B). The *GUS* expression in the *proNnCYP80A:GUS* transgenic plants, however, remained undetectable as in the Milli-Q-treated controls.

In plants, MYC2 is a well-known master regulator in the JA signaling pathway [43]. According to our previously conducted RNA-seq data [36], the *NnMYC2* (NnMYC2, Chr06.g22637) was an early-induced JA responsive gene. Its expression in the lotus lamina was strongly induced by MeJA treatment (Fig. 5D). Similar as the results observed in the MeJA and wounding treatments, transient overexpression of *NnMYC2* in the transgenic *Arabidopsis* leaves significantly enhanced the *proNnCYP80G:GUS* expression, but showed no effect on the *proNnCYP80A:GUS* expression (Fig. 5C). Furthermore, when *NnMYC2* was transiently overexpressed in the lotus petals, the expression of *NnCYP80G* was significantly upregulated, to ~10-fold of the levels in the control (Fig. 5E), while the expression of *NnCYP80A* was not affected. In line with this, the accumulation of aporphine-type BIAs in the petals was significantly improved, with a net increase ~28% at 2 days after infiltration (DAI) (Fig. 5F). In contrast, the total amount of bis-BIAs remained at low levels, accounting to ~1% of the total BIA in the petals.

To check whether the NnMYC2 directly regulates the *NnCYP80G* and *NnCYP80A*, dual luciferase and yeast one-hybrid (Y1H) assays were further conducted. Coinfiltration of NnMYC2 with *proNnCYP80G:LUC* and *proNnCYP80A:LUC* in *N. benthamiana* leaves significantly activated the *proNnCYP80G*- and *proNnCYP80A*-driven LUC expression, by 2.2- and 4.2-fold increase, respectively, against their controls (Fig. 5G and H), indicating the strong activation ability of NnMYC2 on both *NnCYP80G* and *NnCYP80A* promoters. The Y1H assays, however, showed that the NnMYC2 could not bind directly to either *NnCYP80G* or *NnCYP80A* promoters (Fig. 5I).

Taken together, our biochemical results suggested that NnMYC2 can activate both *NnCYP80G* and *NnCYP80A* expression. Transient overexpression of NnMYC2 in the lotus petals, however, activated only the *NnCYP80G* expression, but not *NnCYP80A* expression. The promoter of *NnCYP80A* seems decide that *NnCYP80A* can only be expressed in the lotus plumules, which is not even affected by MeJA and wounding treatments. The NnMYC2 TF seems to act as a very upstream regulator in the JA pathway, which requires to activate other TFs to modulate the *NnCYP80G* and *NnCYP80A* expression.

### NnMYB14 positively regulates the *NnCYP80G* expression and aporphine-BIA biosynthesis

**Figure 6:**
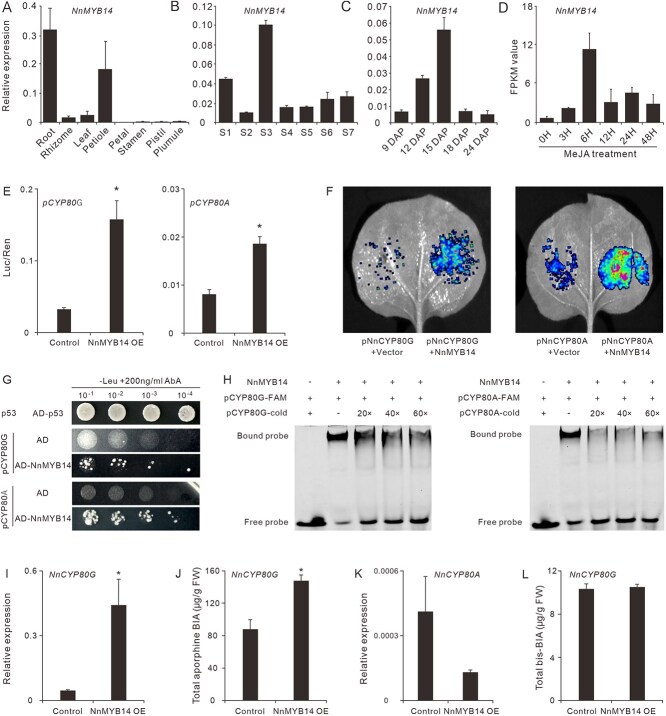
NnMYB14 positively regulates the *NnCYP80G* and the aporphine BIA biosynthesis. (A–C) *NnMYB14* expression in the eight lotus organs (A), in the seven lamina developmental stages (B), in the five plumule developmental stages (C). (D) *NnMYB14* expression in lotus lamina under MeJA treatment. *NnMYB14* expression was marked with FPKM values, according to our previously conducted RNA-seq data. (E) Dual-luciferase assay showing that NnMYB14 could activate both *NnCYP80G* and *NnCYP80A* promoters*.* (F) Transcription activity assay showed that overexpression of *NnMYB14* in *N. benthamiana* leaves significantly promoted both *pNnCYP80G:GUS* and *pNnCYP80A:GUS* expression. (G) Y1H assay showing that the NnMYB14 could physically bind to both *NnCYP80G* and *NnCYP80A* promoters. Numbers of 10^−1^, 10^−2^, 10^−3^, and 10^−4^ indicate the dilution factors of the yeast cultures before spotting on the medium. (H) EMSA determining the direct binding of NnMYB14 to the MBSs in the *NnCYP80G* and *NnCYP80A* promoters. The NnMYB14 dependent mobility shifts were detected, which were competed by the unlabeled cold probe. (I) and (J) Transient overexpression of *NnMYB14* in lotus petals significantly enhanced the *NnCYP80G* expression (I) and the accumulation of aporphine alkaloids (J). (K) and (L) Transient overexpressing *NnMYB14* in lotus petals showed little effect on *NnCYP80A* expression (K) and the accumulation of bis-BIA alkaloids (L). Data are means ± SE (*n* = 3), and asterisks indicate statistically significant differences determined by a two-tailed *t*-test (*P* ≤ 0.05).

Previously, we have identified an R2R3-type MYB TF, NnMYB4 (ID 104610016 or Chr01.g04708), potentially involved in the regulation of BIA biosynthesis in lotus [30]. This MYB TF is clustered together with the *Arabidopsis* AtMYB14 in the phylogenetic tree (Fig. S6) [44], thus it was renamed as NnMYB14. Spatially, *NnMYB14* was highly expressed in the lotus root, rhizome, and lamina organs, while its expression was marginally low in the plumules (Fig. 6A). Developmentally, *NnMYB14* was universally expressed in all the seven leaf developmental stages, while it had the highest expression levels at Stage 3 (Fig. 6B). In the five plumule developmental stages, *NnMYB14* expression was increased in the early stages, peaked at 15 DAP, then decreased in the later stages (Fig. 6C). This was well consistent with the bis-BIA accumulation patterns. In addition, according to our RNA-seq data, the *NnMYB14* is a JA-responsive gene. Its expression was significantly enhanced by the MeJA treatment (Fig. 6D). These results suggest that NnMYB14 is probably involved in the biosynthesis of lotus BIAs.

To further characterize the role of NnMYB14 on lotus BIA biosynthesis, we tested whether NnMYB14 regulates *NnCYP80G* and *NnCYP80A* expression. Initial promoter analysis using PLANTCARE software [45] revealed the presence of potential MYB-binding sites (MBSs, [T/C]AACT[G/A]) in the promoters of *NnCYP80G* and *NnCYP80A* genes (Fig. S7), indicating their direct regulation by MYB TFs. Hence, a dual-luciferase reporter assay was performed to investigate the interaction between NnMYB14 and the promoters of the two genes. Coinfiltration of NnMYB14 along with either *proNnCYP80G:LUC* or *proNnCYP80A:LUC* in *N. benthamiana* leaves significantly enhanced the *LUC* expression, by 2.2 and 3.1 times against the controls (Fig. 6E), indicating the strong ability of NnMYB14 in activating both *NnCYP80G* and *NnCYP80A* promoters. This activation ability was also corroborated by the luciferase luminescence assays (Fig. 6F). Subsequently, to check the direct binding of NnMYB14 to the *NnCYP80G* and *NnCYP80A* promoters, Y1H and electrophoretic mobility shift assays (EMSAs) were further performed. Y1H results showed that yeast cells carrying NnMYB14 and the *NnCYP80G* or *NnCYP80A* promoter could grow on SD/−Leu-deficient medium supplemented with 200 ng/ml aureobasidin A (AbA) (Fig. 6G; Fig. S8), indicating the direct binding of NnMYB14 to both *NnCYP80G* and *NnCYP80A* promoters. The EMSAs revealed that NnMYB14 protein could bind to all the three MBSs in the *NnCYP80G* promoter and the two MBSs in the *NnCYP80A* promoter, and the binding strength decreased gradually after the addition of unlabeled cold probes (Fig. 5H; Fig. S9). These results suggested that NnMYB14 could bind directly to the MBSs in the *NnCYP80G* and *NnCYP80A* promoters and positively activate their expression.

To explore the *in planta* function of NnMYB14, we transiently overexpressed *NnMYB14* on lotus petals by *Agrobacterium-*mediated expression assay. As a result, *NnCYP80G* expression was dramatically elevated (Fig. 6I), and the contents of five aporphine alkaloids in the lotus petals were significantly elevated to 1.84 times of their levels in the controls (Fig. 6J, Fig. S10A). In contrast, *NnMYB14* overexpression showed little effect on the *NnCYP80A* expression, which kept at marginal levels as in the control petals (Fig. 6K), and the concentration of bis-BIAs (liensinine and neferine) remained at low levels (Fig. 6L, Fig. S10B).

Since transient overexpression of either of NnMYC2 or NnMYB14 significantly promoted *NnCYP80G* expression and aporphine BIA biosynthesis, we conducted further NnMYC2 and NnMYB14 coexpression on lotus petals. Transient coexpression NnMYC2 and NnMYB14 on the lotus petals, however, did not show synergistic effects. The expression of *NnCYP80G,* as well as the accumulation of aporphine-type BIAs in the petals coexpression NnMYC2 and NnMYB4, were at the similar levels as those in the petals overexpression either NnMYC2 or NnMYB14 independently (Fig. S11).

### *NnMYB14* is a direct target of NnMYC2

**Figure 7:**
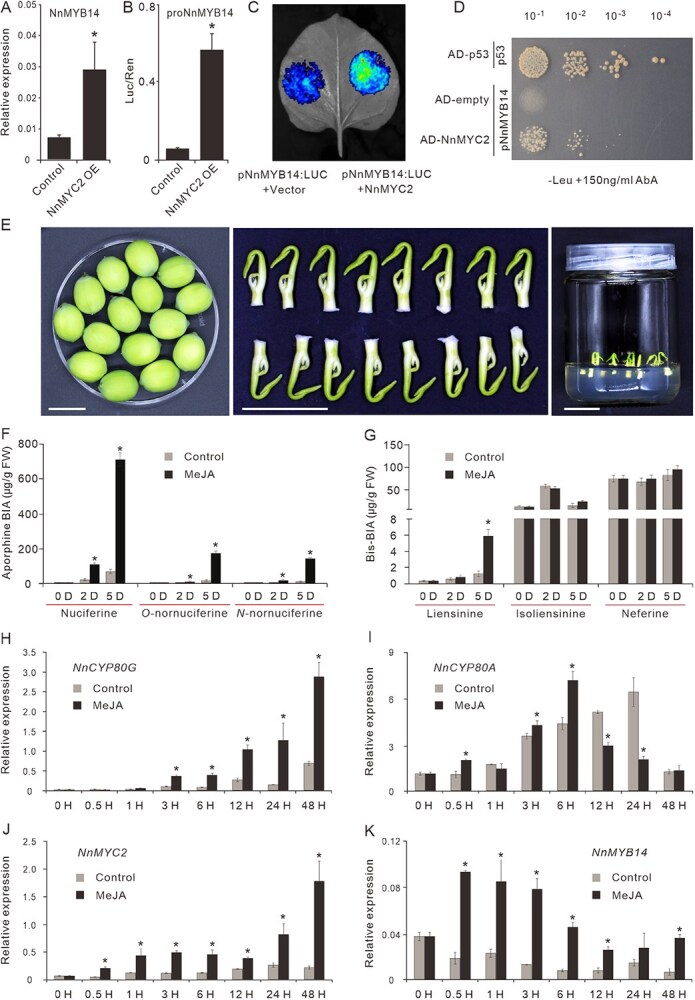
NnMYC2 positively regulates the *NnMYB14* expression and lotus BIA biosynthesis. (A) Transient overexpressing NnMYC2 activated significantly the NnMYB14 expression. (B) and (C) Dual-luciferase assay showed that NnMYC2 could activate the *NnMYB14* promoter-derived luciferase expression. (D) Y1H assay showed that NnMYC2 could bind directly to the *NnMYB14* promoter. (E) MeJA treatment of lotus plumules. Bars represent the length of 1 cm. Plumules were collected from immature lotus seeds at 12 DAP. (F) and (G) The accumulation of major aporphine-BIAs (F) and bis-BIAs (G) in the plumules treated with water (marked in gray) and MeJA (marked in black). (H–K) The relative expression levels of NnCYP80G (H), NnCYP80A (I), NnMYC2 (J), and NnMYB14 (K) in the plumules treated with water and MeJA. Data are means ± SE (*n* = 3), and asterisks indicate statistically significant difference determined by two-tailed *t*-test (*P* ≤ 0.05).

It is worth noting that, in addition to the *NnCYP80G*, the *NnMYC2* overexpression also significantly enhanced *NnMYB14* expression in the lotus petals, indicating the potential regulation role of NnMYC2 on *NnMYB14* expression (Fig. 7A). To test whether NnMYC2 directly regulates *NnMYB14* expression, we conducted further interaction studies on the NnMYC2 and *NnMYB14* promoter. At first, we analyzed the ~1.6-kb promoter region of *NnMYB14* and identified eight potential MYC2-binding sites, including three G-boxes and five E-boxes (Fig. S6). Subsequently, we performed a dual-luciferase assay. Compared with the control (pSAKempty+ *proNnMYB14:LUC*), coinfiltration of NnMYC2 together with the *proNnMYB14:LUC* in *N. benthamiana* leaves significantly promoted *LUC* expression, demonstrating the activation role of NnMYC2 on *NnMYB14* expression (Fig. 7B and C). To check whether NnMYC2 binds directly to *NnMYB14* promoter, a Y1H assay was further conducted. Coexpression of AD-NnMYC2 together with AbAi-*proNnMYB14* enabled their growth on SD/−Leu-deficient medium supplemented with 150 ng/ml AbA (Fig. 7D). These results suggested that NnMYC2 could bind directly to the *NnMYB14* promoter and activate its expression.

As described above, our biochemical experimental evidences have demonstrated the positive regulation role of NnMYC2-NnMYB14 module on both *NnCYP80G* and *NnCYP80A* expression. However, transient overexpression of either *NnMYC2* or *NnMYB14* in the lotus petals promoted only *NnCYP80G* expression and aporphine-BIA biosynthesis. The *NnCYP80A* expression and bis-BIA biosynthesis were hardly affected. Thus, to check whether NnMYC2-NnMYB14 module also regulates *NnCYP80A* expression and bis-BIA biosynthesis *in planta*, we conducted MeJA treatment on lotus plumules, the organ where *NnCYP80A* is specially expressed (Fig. 7E). As a result, the levels of the major aporphine BIAs in the plumules were tremendously elevated to ~10 times their levels in the controls at 120 h after treatment (HAT) (Fig. 7F). In line with the increased aporphine levels, the expression of *NnCYP80G* was markedly upregulated in the MeJA-treated plumules by 1.5- to 8.2-fold of the levels against the controls, since 0.5 HAT (Fig. 7H). In contrast, the MeJA treatment showed relatively moderate effects on the biosynthesis of bis-BIAs. MeJA treatment significantly elevated only the levels of liensinine at 5 days after MeJA treatment, but not for isoliensinine or neferine at all timepoints (Fig. 7G). The expression of *NnCYP80A* was upregulated during the early period of MeJA treatment (0–6 HAT), while it was downregulated at latter timepoints (12–48 HAT) (Fig. 7I). Similar to the results observed on the lotus petals, MeJA treatment on the plumules significantly promoted both *NnMYC2* and *NnMYB14* expression (Fig. 7J and K). Thus, the NnMYC2-NnMYB14 module prominently regulated *NnCYP80G* expression and aporphine-BIA biosynthesis, while it regulated in a much moderate manner the *NnCYP80A* expression and bis-BIA biosynthesis in the lotus plumuls.

## Discussion

BIAs are a diverse class of plant-derived compounds, most of which possess significant medicinal properties [8, 13]. In lotus, BIAs represent the main bioactive constituents in the lamina and plumule tissues, and actualize major therapeutic efficacies such as antioxidant, antiobesity, anticancer, antivirus, and hepato-protection [26, 27]. As a potential resource of future drug innovation, there is an urgent need to unveil the underlying mechanism of lotus BIA biosynthesis and regulation. In this study, we functionally validated two tissue-specific lotus *CYP80* genes, *NnCYP80G* and *NnCYP80A*, and comprehensively characterized their evolution, protein structures, and phenol-coupling activities in the lotus BIAs biosynthesis. Our results revealed that tandem duplication and the subsequent functional divergence of an ancient *CYP80* gene gave rise to the organ-specific accumulation of lotus BIAs. We also characterized an NnMYC2-NnMYB14 module that positively regulates *NnCYP80G* and *NnCYP80A* expression and BIA biosynthesis in lotus. To our knowledge, this is the first report elucidating the organ-specific accumulation of BIAs in the lotus laminae and plumules.

At first, we identified two tissue-specific lotus *P450* genes, *NnCYP80G* and *NnCYP80A*, based on the genome-wide *P450* gene analysis and comprehensive RNA-seq analysis. The two genes were expressed specially in the lotus laminae and plumules, which matched well to the organ-specific accumulation of aporphine BIAs and bis-BIAs in the two organs, respectively (Figs 2 and 3). Tissue-specific expression of CYP80s was also observed in Menispermaceae, which was supposed to drive the BIA diversity in different tissues [20]. It has been shown that gene spatial expression in plants is regulated primarily by the distal region of gene promoters [45, 46]. In the current study, our evidences showed that the tissue-specific expression of the two genes was also determined by their promoters. Histochemical GUS analysis of the *proNnCYP80G:GUS* and *proNnCYP80A:GUS* transgenic *Arabidopsis* revealed that the *proNnCYP80G*-driven *GUS* was expressed particularly in the chlorophyll-rich tissues, while the *porNnCYP80A*-driven *GUS* was primarily expressed in the young seeds. Despite sharing high amino acid sequence identity (79.7%) in the coding region, their promoter sequences displayed relatively low identity (47.1%). In addition to the majority shared *cis*-acting regulatory elements (CAREs), the *NnCYP80G* and *NnCYP80A* promoters also comprise gene-specific elements. For example, the *NnCYP80G* promoter contains especially Box II, TATC-box, TCA, and W-box elements, while the *NnCYP80A* promoter contains particularly LTR, F-box, MeJA, and Auxin-responsive elements (Fig. S12). These disparate *cis*-elements contributed possibly to the tissue-specific expression of *NnCYP80G* and *NnCYP80A*. It should be noticed that the expression of *NnCYP80A* in the lotus leaves was not even induced by MeJA, wounding, and *NnMYC2* overexpression treatments (Fig. 5). In contrast, the expression of *NnCYP80G* in the plumules was readily induced by MeJA treatment, and the accumulation levels of aporphine-BIAs were significantly enhanced.

Subsequently, we characterized *in planta* the phenol-coupling activities of NnCYP80G and NnCYP80A. Recently, based on the yeast platforms, two studies have independently characterized the enzymatic activities of NnCYP80A and NnCYP80G in BIA biosynthesis [31, 34]. Both studies revealed that NnCYP80A and NnCYP80G preferentially took the (*R*)-*N*-methylcoclaurine rather than (*S*)-*N*-methylcoclaurine substrate, and catalyzed the biosynthesis of a proaporphine alkaloid glaziovine and a bis-BIA nelumboferine, respectively. In contrast, another recent study reported that both of the proteins also consumed *S*-enantiomer substrates [33]. They showed experimentally that NnCYP80A converted (*S*)-*N*-methylcoclaurine and the mixture of (*R*)- and (*S*)-*N*-methylcoclaurine into different bis-BIA products. In addition to (*R*)-*N*-methylcoclaurine, NnCYP80G also efficiently consumed (*S*)-*N*-methylcoclaurine, reticuline, and coclaurine substrates, leading to the production of various aporphine BIAs. In this study, we overexpressed NnCYP80G and NnCYP80A in *N. benthamiana* through a previously reported *Agrobacterium*-mediated expression system [47]. Our results revealed the obvious *R*-enantiomer substrate preference for both NnCYP80G and NnCYP80A, which was consistent with the data reported by professors Martin and Facchini [31, 34]. Among the five supplied substrates, NnCYP80A consumed only (*R*)-*N*-methylcoclaurine, and converted it into bis-BIA of nelumboferine, whereas NnCYP80G efficiently took both (*R*)-*N*-methylcoclaurine and (*R*)-reticuline, and converted them into a proaporphine of glaziovine and an aporphine of corytuberine, respectively. It is the first we reported NnCYP80G converting (*R*)-reticuline substrate into the aporphine product of corytuberine.

Moreover, we demonstrated that the JA-responsive NnMYC2-NnMYB14 module positively regulates *NnCYP80G* and *NnCYP80A* expression and lotus BIA biosynthesis. MYC2 is a well-known plant master regulator in the JA signaling pathway [43, 48–50]. Our results showed that lotus MYC2 activated strongly both *NnCYP80G* and *NnCYP80A* promoters, but could not bind directly to either of their promoters. Obviously, there are other TFs downstream of the NnMYC2 in regulation of lotus BIA biosynthesis. Interestingly, transient overexpression of NnMYC2 in the lotus petals promoted not only *NnCYP80G* and *NnCYP80A* expression, but also significantly *NnMYB14* expression. NnMYB14 is an R2R3-type MYB TF highly homologous to *Arabidopsis* MYB14 (Fig. S5) [44]. Previous studies have shown that the AtMYB14 is involved in plant response to various biotic and abiotic stresses [51–53]. In this study, our results showed that NnMYB14 can positively regulate lotus BIA biosynthesis by directly binding to both *NnCYP80G* and *NnCYP80A* promoters and activating their expressions. Markedly, dual-luciferase and Y1H assays revealed that NnMYC2 could directly bind directly to the *NnMYB14* promoter and activate its expression. To our knowledge, this is the first report showing AtMYB14-type R2R3 MYB involved in the regulation of plant alkaloid biosynthesis.

It should be noted that MeJA and wounding treatments, as well as transient overexpression of *NnMYC2* or *NnMYB14* in the lotus petals significantly upregulated only *NnCYP80G* but not *NnCYP80A* expression. This is probably due to the tissue-specific characteristics of the two genes. However, MeJA treatment on the lotus plumules significantly elevated not only *NnMYC2* and *NnMYB14* expression, but also *NnCYP80G* and *NnCYP80A* expression. The biosynthesis of all aporphines and the bis-BIA of liensinine were largely induced. Thus, both *NnCYP80G* and *NnCYP80A* are targets of the NnMYC2-NnMYB14 module in the lotus plumules, although *NnCYP80G* is more dramatically upregulated than *NnCYP80A*. On the other hand, *NnCYP80A* is not expressed in lotus lamina and leaf organs, and its expression is not even inducible by the MeJA and wounding treatments. In contrast, although the background expression of *NnCYP80G* in the lotus plumules is marginally low, it is readily induced by MeJA treatment.

**Figure 8:**
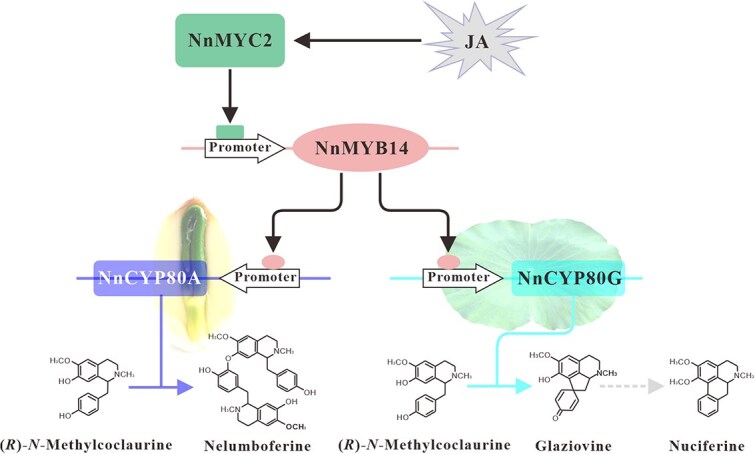
Proposed models for the underlying mechanisms of organ-specific accumulation of aporphines and bis-BIAs in the lotus plumules and laminae, respectively. Environmental cues, including insect bite (mechanical wounding), elevate bioactive JA and induce the expression of *NnMYC2*, a master regulator of secondary metabolite biosynthesis. NnMYC2 binds directly to the promoter of *NnMYB14* and positively regulate its expression. Subsequently, the NnMYB14 binds directly to the *NnCYP80G* and *NnCYP80A* promoters and upregulate their expression. The *NnCYP80G* and *NnCYP80A* are specially expressed in the lotus laminae and plumules, respectively. NnCYP80A could convert (*R*)-*N*-methylcoclaurine substrate into bis-BIAs, whereas NnCYP80G catalyzes the formation of pronuciferine and aporphines.

Based on the presented results, we propose a model to depict how NnMYC2-NnMYB14-NnCYP80G/NnCYP80A modules regulate the organ-specific deposition of BIAs in lotus (Fig. 8). Environmental cues, such as insect bite (mechanical wounding), elevate bioactive JAs and trigger *NnMYC2* expression. NnMYC2 binds directly to the *NnMYB14* promoter and activates its expression. Subsequently, NnMYB14 positively regulates the *NnCYP80G* and *NnCYP80A* expression by direct binding to their promoters. Eventually, the organ-specific expression and the functional divergence of *NnCYP80G* and *NnCYP80A* results in the specific accumulation of aporphine BIAs in the lotus laminae and bis-BIAs in the plumules. However, the NnMYC2-NnMYB14 module seems to be a relatively weak regulator for *NnCYP80A* in the lotus plumules. Further studies are required to investigate novel pathways regulating *NnCYP80A* expression and bis-BIA biosynthesis.

## Materials and methods

### Plant materials

Two lotus cultivars, cv. ‘Jianxuan 17’ and cv. ‘Qiuxing’, were used in this study. They were rhizome-propagated and cultivated in pots (diameter, 40 cm; height, 40 cm) at the Wuhan Botanical Garden, Chinese Academy of Sciences (Wuhan, China). All pots were set outdoors on the ground and managed with routine water and fertilizer supply as previously described [30]. The temporal and spatial accumulation of BIAs and the expression patterns of *CYP80* genes in this study were determined in cv. ‘Jianxuan 17’, while transient overexpression of *CYP80* genes was conducted in cv. ‘Qiuxing’.

*Arabidopsis* (Col-0) and tobacco (*N. benthamiana*) plants for transgenic overexpression analyses were grown in our cultivation room under the conditions of 25/23°C day/night temperature, 16 h photoperiod, 250 μmol m^−2^ s^−1^ light intensity, and 40% relative humidity.

### Methyl jasmonate treatment of lotus plumules

Lotus seeds were collected at 12 days postpollination (DAP), and surface-sterilized in 75% ethanol for 3 min. Then, plumules were isolated aseptically with surgical blades and forceps, transferred to culture medium (one-half MS, 30 g/l Sucrose, 8 g/l Agar, and pH = 5.8), and incubated in darkness at 28°C. For MeJA treatment, plumules were grown on one-half MS medium supplemented with 100 μM MeJA, and maintained under dark conditions. Samples were collected at 0, 0.5, 1, 3, 6, 12, 24, 48, and 120 h post-treatment. All assays were repeated three times, with three biological replicates.

### Mining and analysis of lotus P-450 genes

Hidden Markov model (HMM) was used to screen lotus P-450 family genes containing the typical P-450 domain (PF00067) in our unpublished lotus T2T genome (Sun *et al*., unpublished data), with the *E*-value threshold of 1.0e-05. Full-length protein sequences of all lotus P-450 s and selected CYP80 references were aligned with MUSCLE [54], and neighbor-joining (NJ) phylogenetic trees were constructed in MEGA7 [55] with 1000 replicates. Trees were visualized with the FigTree software (V1.4.2). The reference CYP80 proteins used for tree construction were retrieved from six plant species with the following accession numbers: *C. japonica*, CjCYP80B2 (Q9FXW4.1) and CjCYP80G2 (A8CDR5.1); *B. stolonifera*, BsCYP80A (P47195.1); *Hyoscyamus niger*s, HnCYP80F1 (ABD39696.1); *Thalictrum flavum*, TfCYP80B (AAU20767.1); *P. somniferum,* PsCYP80B1 (AAF61400.1); and *E. californica*, EcCYP80B1 (O64900.1).

For evolutionary analysis of plant CYP80s, 10 plant species were selected, including representatives of mosses (*P. patens*), ferns (*S. moellendorffii*), gymnosperms (*P. taeda*), the most basal extant flowering plant (*A. trichopoda*) [56], monocots (*O. sativa* and *M. acuminata*), dicots accumulating BIAs (*N. colorata*, *N. nucifera*, and *S. lycopersicum*), and dicots accumulating no BIA (*A. thaliana*). The CYP80 or CYP80-like genes of these species were predicted based on BLASTX analysis with a threshold value of 1e-5 in each genome using *CjCYP80G2* as a query. Collinearity among lotus *P-450* genes was analyzed using the MCScanX feature in TBtools [57].

### Analysis of *NnCYP80A* and *NnCYP80G* promoter activities in transgenic *Arabidopsis*

Approximately 1.5 kb genomic sequence regions upstream of the *NnCYP80G* and *NnCYP80A* translation start sites were PCR-amplified and cloned into the pBI101 vector for *proCYP80G/80A:GUS* through the *PstI* and *XbaI* restriction sites. The recombinant vectors were subsequently introduced into *A. tumefaciens* (GV3101), and used for *Arabidopsis* (Col-0) transformation via floral dipping. Mechanical wounding treatments were conducted on the rosette leaves of 4-week-old transgenic plants, with 1 ml sterile syringes. MeJA treatments were performed by spraying 100 μM MeJA solutions on transgenic *Arabidopsis* plants at 7 days postgermination. Samples were collected 24 h after wounding treatments or 72 h post-MeJA treatments for GUS staining.

GUS activity of transgenic *Arabidopsis* plants was analyzed with the GUS Stain Kit (Zhongke real-times, Beijing, China). Plant samples, including plantlets, leaves, inflorescences, and siliques, were submerged and vacuum-infiltrated for 20 min in the GUS buffer containing 5-bromo-4-chloro-3-indolyl-b-D-glucuronic acid (X-gluc) substrate, and subsequently incubated in darkness at 37°C for 24 h. After staining, the samples were dehydrated with 70% ethanol for three times to remove chlorophyll. Images were taken with a research stereo microscope (Nikon SMZ25, Tokyo, Japan). Primers used in the study are listed in Table S1.

### Quantitative real-time PCR

Quantitative real-time PCR was conducted as previously described [58]. Briefly, collected lotus and *Arabidopsis* samples were immediately frozen with liquid nitrogen and ground into fine powder using chilled mortar and pestles. Total RNA was extracted with the total RNApure Reagent (Zoman Biotech, Beijing, China), and the first-strand cDNA was synthesized using the TransScript® II One-Step gDNA Removal and cDNA Synthesis SuperMix (TransGen, Beijing, China). Quantitative real-time PCR was performed using SYBR® Premix Ex Taq™ II (Takara, Dalian, China) in a 15 μl reaction mixture containing 7.5 μl of 2 × SYBR TB Green Premix Ex TaqII, 0.3 μl of ROX Reference Dye, 100 μM of each primer, and 50 ng of template cDNA. The lotus *Actin2* and *Arabidopsis Actin* genes [35] were used as the internal references, and the relative expressions of target genes were calculated with three biological and three technical replicates using the 2^−ΔΔCt^ method [59]. Primers used in this study are listed in Table S1.

### Subcellular localization assay

Full-length coding sequences of *NnCYP80G* and *NnCYP80A* without termination codons were PCR-amplified and inserted into the pMDC83 vector for construction of CaMV 35S:NnCYP80G-GFP and 35S:NnCYP80A-GFP vectors. The recombinant constructs were transformed into the *A. tumefaciens* strain GV3101. *Agrobacterium tumefaciens* carrying the Mcherry-ER marker plasmid was used as positive control. The transformed *Agrobacterium* cells were collected and resuspended in infiltration buffer (10 mM MES buffer, 10 mM MgCl_2_, 200 mM acetosyringone) to OD_600_ = 0.6. Prior to tobacco infiltration, *Agrobacterium* cells carrying the Mcherry-ER plasmid and 35S:NnCYP80G-GFP (or 35S:NnCYP80A-GFP) construct were mixed in a 1:1 volume ratio. Agroinfiltration was conducted in 5- to 6-week-old *N. benthamiana* plants using a 1-ml needleless syringe. Fluorescence signals were captured 2 days later using the Zeiss Confocal Fluorescence Microscope (LSM710 Meta, Carl Zeiss). The primers used for pMDC83 vector construction are listed in Table S1.

### Transient overexpression of lotus genes in *N. benthamiana* and lotus

Full-length coding sequences of target genes were PCR-amplified and cloned into pSAK277 vector under the 35S promoter. For *NnCYP80G* and *NnCYP80A* overexpression in *N. benthamiana* leaves, *A. tumefaciens* carrying *NnCYP80G* (or *NnCYP80A*) and *NnCPR* expression vectors were individually collected and resuspended to a final concentration of OD_600_ = 0.3, and then mixed with 1:1 volume ratio for agroinfiltration.

To test the enzyme activities of NnCYP80A and NnCYP80G, BIA substrates were infiltrated into the same area of agroinfiltration 4 days postinfiltration, and treated leaves were sampled 24 h later for BIA analysis. Transient overexpression of *NnMYC2*, *NnMYB14*, *NnCYP80G*, and *NnCYP80A* in the lotus petals was performed the same as previously described [60]. All experiments were repeated at least three times with three biological replicates.

### Lotus alkaloid extraction and analysis

Alkaloid was extracted according to our previously reported protocol [24]. Briefly, the samples were finely ground into powder with liquid nitrogen, and ~50 mg of powder was transferred to a 2-ml Eppendorf tube and extracted with 1 ml of extract solution (Methanol-H_2_O-HCl, 50:45:5, v/v). The mixture was sonicated for 30 min, and centrifuged at 10 000 rpm at 4°C for 10 min. The supernatant was transferred to a new 2-ml Eppendorf tube, and the pellet was extracted again with 1 ml extract solution. The two supernatants were then combined and set to the volume of 1.5 ml with the extraction buffer.

The alkaloid extracts were analyzed with high-performance liquid chromatography (HPLC) coupled to a high-resolution Q-TOF Mass Spectrometer (6350 Q-TOF LC/MS, Agilent Technologies, Santa Clara, CA, USA). Alkaloid separation was performed with an ACQUITY UPLC® CSH™ C18 column (2.1 × 150 mm, 1.7 μm, Waters, USA). The mobile phase used for HPLC elution consisted of 0.1% (v/v) formic acid aqueous solution (mobile phase A) and 0.1% (v/v) formic acid acetonitrile (mobile phase B), with a flow rate of 0.3 ml/min. The gradient conditions were as follows: 0–1 min, 5% B; 1–16 min, 5%–35% B; 16–17 min, 35%–100% B; 17–22 min, 100% B; 22–24 min, 100%–5% B; and 24–30 min, 5% B. Mass spectra were acquired in the positive ion mode with capillary voltage at 3.5 kV, current 7 μA, spray voltage 500 V, nitrogen flow 5 l/min, atomizer 45 psi, sheath temperature 350°C, cone hole air flow 11 l/min, and a scan range of *m*/*z* 100–1700.

### Dual-luciferase reporter assay

Promoter regions ~1.5 kb upstream *NnCYP80G* and *NnCYP80A* translation start codon (ATG) were PCR-amplified and cloned into the dual-luciferase (LUC) reporter gene expression vector pGreen0800-LUC to form *proNnCYP80G:LUC* and *proNnCYP80G:LUC* constructs, while complete *NnMYC2* (Chr06.g22637) or *NnMYB14* coding sequence (CDS) was cloned into pSAK277 vector to form the TF expression vectors. Dual-luciferase assay steps, including *Agrobacterium* transformation, agroinfiltration, and the measurements of Firefly luciferase (F-Luc) and Renilla luciferase (R-Luc) activity, were conducted as previously described [30]. Primers used for vector cloning are listed in Table S1.

### Yeast one-hybrid assay

Y1H assay was performed following the user manual of Matchmaker® Gold Yeast One-Hybrid Library Screening System (Clonteck, USA). Promoters of *NnCYP80A*, *NnCYP80G* and *NnMYB14*, with lengths of 848, 1421, and 1587 bp upstream of the gene translation start codon (ATG), were cloned into pAbAi vectors to generate the bait-reporter yeast strains, while the *NnMYC2* and *NnMYB14* full-length CDSs were cloned into pGADT7 vector to generate pGADT7:NnMYC2 and pGADT7:NnMYB14 constructs. The minimal inhibitory concentration of Aureobasidin A (200 ng/ml) was determined with different bait-reporter yeast strains carrying the empty pGADT7 AD vector. To evaluate whether the TFs interact with targeted promoters, TF plasmids were transformed into Y1HGold bait strains carrying promoter vectors, and cultured on SD/−Leu containing 200 ng/ml AbA. Y1HGold bait strains transformed with empty pGADT7 and Y1HGold (p53-AbAi) transformed with pGADT7-p53 were set as negative and positive controls, respectively. Primers used for vector cloning were listed in Table S1.

### Electrophoretic mobility shift assay

To produce recombinant NnMYB14 and NnMYC2 proteins, their full-length CDS were cloned into the pET-32α vector. The resulting plasmids were then transformed into BL21 cells. For induction of recombinant protein expression, cell cultures were grown at 37°C in LB medium until reaching an OD_600_ of 0.8. Protein expression was induced by adding isopropyl β-D-1-thiogalactopyranoside (IPTG, Sigma, USA) to a final concentration of 0.5 mM and incubating at 37°C for 3 h. Tagged proteins were purified using the His protein purification kit (P2229S, Beyotime, Shanghai, China) and checked by SDS-PAGE gel electrophoresis.

For EMSA, DNA probes with or without 5′ FAM (Fluorescein) labeling were synthesized (Tsingke, Beijing, China). The probes (Table S1) were designed to include putative MYB-binding sites in the CYP80A/G promoters and the MYC-binding site in the MYB14 promoter. DNA-binding reactions were performed at room temperature for 30 min in a system containing EMSA binding buffer (GS009-1, Beyotime, Shanghai, China), 100 nM FAM-labeled DNA, and indicated amounts of proteins, with or without unlabeled cold probe competitors. During the competitive experiment, cold probes were added in an excess molar ratio (20, 40, and 60 times) than FAM-labeled probes. DNA-protein complexes were resolved by PAGE gel electrophoresis, followed by the detection of band shifts using a fluorescent image analyzer (Typhoon FLA 9000, GE Healthcare Bio-Sciences, Pittsburgh, PA, USA).

## Supplementary Material

Web_Material_uhaf283

## Data Availability

All data needed to evaluate the conclusions in the paper are present in the paper and/or the Supplementary materials.
